# The causal relationship between gut microbiota and nine infectious diseases: a two-sample Mendelian randomization analysis

**DOI:** 10.3389/fimmu.2024.1304973

**Published:** 2024-07-10

**Authors:** Song Wang, Fangxu Yin, Wei Sun, Rui Li, Zheng Guo, Yuchao Wang, Yiyuan Zhang, Chao Sun, Daqing Sun

**Affiliations:** ^1^ Department of Pediatric Surgery, Tianjin Medical University, General Hospital, Tianjin, China; ^2^ Department of Reproductive Endocrinology, Second Hospital of Shandong University, Jinan, China; ^3^ Department of Orthopedic Surgery, Tianjin Medical University General Hospital, Tianjin, China

**Keywords:** gut microbiota, infectious diseases, causality, GWAS, Mendelian randomization

## Abstract

**Background:**

Evidence from observational studies and clinical trials has associated gut microbiota with infectious diseases. However, the causal relationship between gut microbiota and infectious diseases remains unclear.

**Methods:**

We identified gut microbiota based on phylum, class, order, family, and genus classifications, and obtained infectious disease datasets from the IEU OpenGWAS database. The two-sample Mendelian Randomization (MR) analysis was then performed to determine whether the gut microbiota were causally associated with different infectious diseases. In addition, we performed reverse MR analysis to test for causality.

**Results:**

Herein, we characterized causal relationships between genetic predispositions in the gut microbiota and nine infectious diseases. Eight strong associations were found between genetic predisposition in the gut microbiota and infectious diseases. Specifically, the abundance of class *Coriobacteriia*, order *Coriobacteriales*, and family *Coriobacteriaceae* was found to be positively associated with the risk of lower respiratory tract infections (LRTIs). On the other hand, family *Acidaminococcaceae*, genus *Clostridiumsensustricto1*, and class *Bacilli* were positively associated with the risk of endocarditis, cellulitis, and osteomyelitis, respectively. We also discovered that the abundance of class *Lentisphaeria* and order *Victivallales* lowered the risk of sepsis.

**Conclusion:**

Through MR analysis, we found that gut microbiota were causally associated with infectious diseases. This finding offers new insights into the microbe-mediated infection mechanisms for further clinical research.

## 1 Introduction

Infections such as pneumonia and gastrointestinal infections are the most common infections in hospitalized patients (1). Statistically, these infections account for more than 20% of deaths globally, with 245,000 sepsis cases occurring in the United Kingdom (UK) alone annually (2, 3). Owing to antibiotic resistance, an aging population, and emerging pathogens, the infection-induced disease burden is expected to rise, making the identification of the factors that can modify these illnesses essential (4–6). Generally, severe bacterial infections are believed to be caused by the invasion of the blood and tissues by pathogenic microorganisms, resulting in tissue necrosis and even host death (7). Furthermore, with advancements in sepsis research in recent years, it has been found that uncontrolled infection may lead to dysregulation of the host’s immune response. At the same time, excessive immune response results in the secretion of a multitude of cytokines, leading to organ dysfunction and, ultimately, host death (8–10). Therefore, effective prevention and treatment of serious infectious diseases has become critical.

In a healthy host, the gut microbiota regulate various homeostasis mechanisms, including immune function and gut barrier protection (11, 12). Mechanisms of gut microbiota leading to infectious diseases, including allowing the expansion of pathogenic gut bacteria, primes the immune system to produce a robust pro-inflammatory response, thus reducing the production of beneficial microbial products, such as short-chain fatty acids (13–15). Furthermore, gut microbiota interact with infectious diseases. On the one hand, susceptibility to infectious diseases may be aggravated by intestinal micro-ecological disorders. Under certain conditions, intestinal bacteria can directly invade peripheral blood through intestinal mucosa. They could also enter distant organs via the “gut–organ” axis, causing bacterial translocation and eliciting systemic inflammatory responses. Further illness progression can lead to organ dysfunction (16). On the other hand, severe infection could also cause alterations in the human intestinal microenvironment, resulting in the imbalance of intestinal flora and the release of inflammatory factors, damaging the intestinal mucosal barrier and further aggravating the disease (17). Although an increasing number of studies has associated gut microbiota with infectious diseases, the causal relationship between the two remains unclear.

In recent years, Mendelian randomization (MR) analysis, a statistical approach for investigating causal relationships, has been mainly applied to the causal inference of epidemiological diseases. Since alleles follow the random allocation principle, this impact is not affected by confounding factors and reverse causation in traditional epidemiological research (18). The publication of large-scale genome-wide association study (GWAS) data has resulted in the availability of a substantial number of reliable genetic variants for MR studies (19). As a result, this study analyzed the causal relationship between gut microbiota and infectious diseases through the MR analysis, providing useful insights into the clinical treatment of infectious diseases.

## 2 Materials and methods

### 2.1 Study population

**Figure 1:**
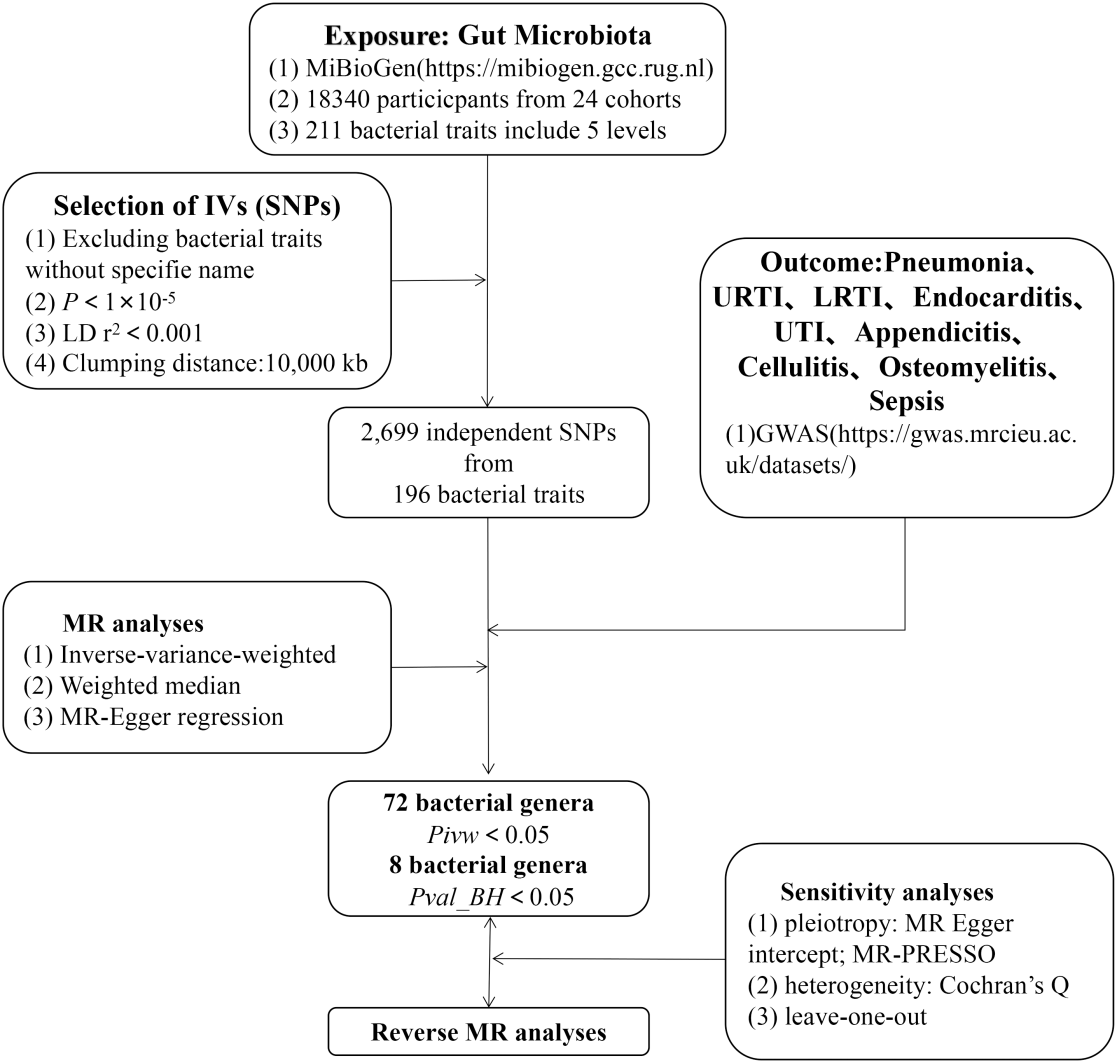
The study design of the present MR study of the associations of gut microbiota and sepsis. LD, linkage disequilibrium, which used to measure the correlations between SNPs; IVW, inverse-variance-weighted, the main analyses to evaluate the relationship between exposure and outcome; MR-PRESSO, Mendelian Randomization Pleiotropy RESidual Sum and Outlier, a method test the pleiotropic biases in the SNPs and correct the pleiotropic effects; MR, Mendelian randomization; SNPs, single-nucleotide polymorphisms, as instrumental variables for the exposures and outcomes.

As shown in **Figure 1**, we used a two-sample MR (TSMR) approach to characterize the causal relationship between the intestinal microbiome and infectious diseases and finally conducted quality control tests, including the heterogeneity and gene pleiotropy tests, to verify the reliability of the results.

The gut microbiota, which is investigated in the context of human genetics by MiBioGen, an international consortium, was the primary exposure factor for our study (20). Herein, the human gut microbiota GWAS data, encompassing 18,340 individuals from 24 population cohorts, was used. A total of 196 bacterial groups (including 9 phyla, 16 classes, 20 orders, 32 families, and 119 genera) were included after excluding 15 genera with no specific species names.

Our primary outcomes were various infectious diseases with GWAS datasets from the UK Biobank project (21), a prospective cohort study that collected deep genetic and phenotypic data on approximately 500,000 individuals across the UK. Each participant had a wealth of phenotypic and health-related information. Genome-wide genotype data were collected from all participants by linking health and medical records to provide follow-up information. Pneumonia, upper respiratory tract infections (URTIs), lower respiratory tract infections (LRTIs), endocarditis, urinary tract infections (UTIs), appendicitis, cellulitis, osteomyelitis, and sepsis were among the infectious diseases evaluated. Information on exposure and outcome factor data is presented in **Supplementary Table 1**.

### 2.2 Single-nucleotide polymorphisms selection

Here, single-nucleotide polymorphisms (SNPs) significantly associated with the relative abundance of 196 gut microbiota were selected as available instrumental variables (IVs). According to previous research, including multiple IVs can enhance the interpretation of exposure variation and improve the accuracy and reliability of analysis results. As a result, to ensure the independence of the included SNPSs, this study selected IVs based on the results of association analysis (with *p* < 1×10^-5^ as the significance threshold), set the linkage disequilibrium criteria (with *R* ^2^ < 0.001) and genetic distance (with 10,000 kb), and excluded highly correlated SNPs (22). Finally, SNPs associated with the relative abundance of gut microbiota were projected into the GWAS data on infectious diseases and the corresponding statistical parameters were retrieved. To align the effect exposure and outcome values with the same effect allele, the data were unified based on the statistical parameters of the same site in the relative abundance of gut microbiota and GWAS results of infectious diseases.

### 2.3 Research design

When using SNPs as IVs in MR analysis, three key assumptions should be met to better estimate the causal effects: (1) The IVs must be closely related to exposure factors; (2) the IVs should not be related to confounding factors; and (3) the IVs should only affect the results through exposure and not by any other means.

### 2.4 Statistical analysis

In this study, Inverse variance weighted (IVW), MR-Egger, Weighted Median (WME), Simple Mode (SM), and Weighted Mode (WM) were used to estimate the causal effect. The IVW method presumes that all genetic variants are valid. The IVW approach employs the ratio method to calculate the causal effect size of individual IVs and obtains the total effect size by aggregating each estimate for weighted linear regression (23). The primary distinction between the MR-Egger and the IVW methods is that the former considers the existence of the intercept term in regression analysis (24). The WME approach takes advantage of all available genetic variants’ intermediate effects. An estimate (25) was obtained by weighting the inverse variance of each SNP’s correlation with the outcome. The SM and WM methods are modality-based approaches, and modality-based estimation models aggregate SNPs with similar causal effects and return the estimates of causal effects for most cluster SNPs. The influence of each SNP on the cluster was weighted by WM per the inverse variance of its resulting effect.

Given that the IVW approach is more efficient than the other four MR methods, it was used herein as the preferred causal effect estimation method. Additionally, the beta values obtained in the results were converted into odds ratios (OR), and the 95% confidence interval (CI) was calculated to better explain the results. To verify whether the results were “false positives” due to multiple tests, we used the Benjamini–Hochberg (BH) method under the false discovery rate (FDR) standard to correct the MR results for different classifications of gut microbiome (phyla, class, order, family, and genus); the calculation formula is FDR(*i*) = *p*(*i*)*m/*i*, specifically, all *p*-values are arranged in ascending order, where *p*-values are denoted as *p*, the serial number of *p*-values is denoted as *i*, and the total number of *p*-values is denoted as *m* (26). Using the *F* statistic to test IV strength, the association of effect estimates that test causation may be affected by weak instrumental bias. The *F* statistic is calculated as follows: *F* = *R* ^2^ (*N−K−*1)/*k* (1−*R* ^2^), where *R* ^2^ = variance (per gut microbiome) interpreted by IV, and *n* = sample size. The *R* ^2^ is estimated from the minor allele frequency (MAF) and *B*-value using the following equation: *R* ^2^ = 2 × MAF × (1−MAF) × *b*2 (27).

Additionally, we included sensitivity analysis, heterogeneity level test, and gene pleiotropy test in quality control to further test the stability and reliability of the results. For sensitivity analysis, the residual one method was used, and the combined effect value of the remaining SNPs was determined by sequentially deleting single SNP to evaluate the impact of each SNP on the results. The heterogeneity test was performed to assess the heterogeneity of SNPs. The SNP measurement error caused by experimental conditions and population analysis, among other factors, could lead to bias in estimating causal effects (28). Using the intercept term of the MR-Egger regression, the horizontal gene pleiotropy test assesses whether IVs affect outcomes by other means apart from exposure (29). Potentially abnormal SNPs were identified through the Mendelian Randomization Multi-Effect Residual and Outlier (MR-PRESSO) (30) and leave-one-out methods (31). Finally, we performed reverse MR to analyze whether there was a reverse causality between infectious diseases and meaningful gut microbiota. The MR Analysis and quality control for this study were analyzed using version 4.0.3 R and version 0.5.6 TwoSampleMR packages.

## 3 Results

### 3.1 TSMR analysis

**Figure 2:**
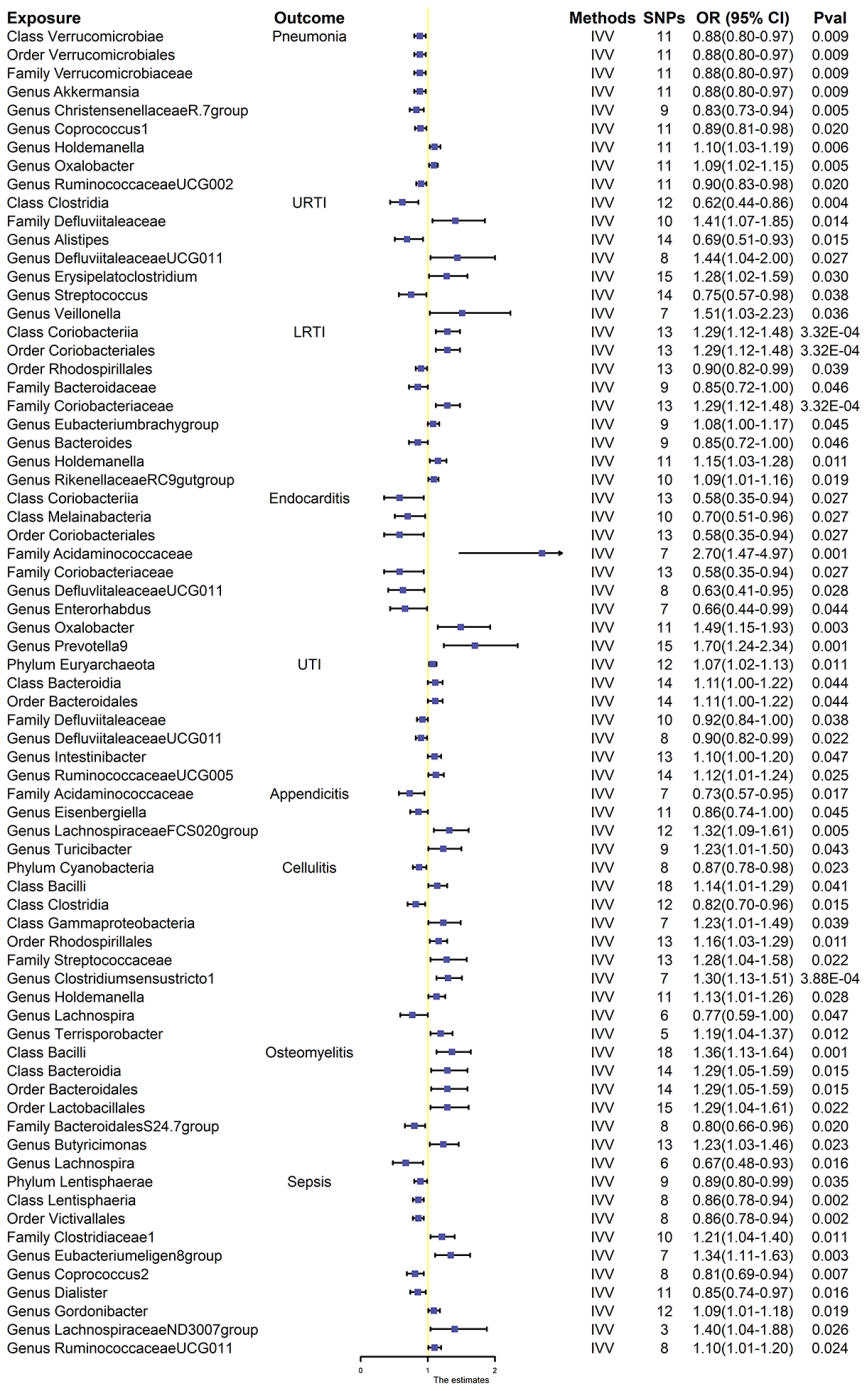
Effect estimates of the association between meaningful gut microbiota and infectious disease risk in IVW analysis. SNPs, single-nucleotide polymorphisms, as instrumental variables for the exposures and outcomes; OR, odds ratio; CI, confidence interval; URTI, upper respiratory tract infection; LRTI, lower respiratory tract infection; UTI, urinary tract infection.

The results of the 196 gut microbiota examined in relation to infectious disease are presented in **Supplementary Table S2**. The *F*-statistics for the gut flora ranged between 14.58 and 88.42 (all meeting the >10 threshold), implying that they are unlikely to be impacted by weak instrumental bias (**Supplementary Table S3**). Briefly, we identified 72 genera associated with infectious disease risk (**Figure 2**). However, after rigorous BH correction, only eight gut microbiota showed stability in their association with infectious diseases (**Table 1**).

**Table 1:** Effect estimates of the association between meaningful gut microbiota and infectious disease risk in MR analysis.

Gut microbiota	Outcome	SNPs	Methods	OR (95% CI)	*p*-value	*p_FDR_*
Class *Coriobacteriia*	LRTI					
		13	MR-Egger	1.28 (0.74–2.22)	0.401	
		13	Weighted median	1.28 (1.05–1.55)	0.012	
		13	IVW	1.29 (1.12–1.48)	3.32E-04	0.005
		13	Simple mode	1.26 (0.91–1.73)	0.187	
		13	Weighted mode	1.26 (0.92–1.71)	0.176	
Order *Coriobacteriales*	LRTI					
		13	MR-Egger	1.28 (0.74–2.22)	0.401	
		13	Weighted median	1.28 (1.06–1.54)	0.010	
		13	IVW	1.29 (1.12–1.48)	3.32E-04	0.007
		13	Simple mode	1.26 (0.94–1.67)	0.147	
		13	Weighted mode	1.26 (0.92–1.71)	0.177	
Family *Coriobacteriaceae*	LRTI					
		13	MR-Egger	1.28 (0.74–2.22)	0.401	
		13	Weighted median	1.28 (1.07–1.53)	0.007	
		13	IVW	1.29 (1.12–1.48)	3.32E-04	0.011
		13	Simple mode	1.26 (0.93–1.69)	0.160	
		13	Weighted mode	1.26 (0.92–1.72)	0.184	
Family *Acidaminococcacea*e	Endocarditis					
		7	MR-Egger	0.73 (0.14–3.77)	0.719	
		7	Weighted median	1.67 (0.82–3.42)	0.159	
		7	IVW	2.70 (1.47–4.97)	0.001	0.045
		7	Simple mode	1.58 (0.61–4.05)	0.382	
		7	Weighted mode	1.60 (0.66–3.88)	0.341	
Genus *Clostridiumsensustricto1*	Cellulitis					
		7	MR-Egger	1.34 (0.96–1.87)	0.145	
		7	Weighted median	1.25 (1.01–1.54)	0.036	
		7	IVW	1.30 (1.13–1.51)	3.88E-04	0.046
		7	Simple mode	1.25 (0.94–1.65)	0.173	
		7	Weighted mode	1.24 (0.97–1.57)	0.132	
Class *Bacilli*	Osteomyelitis					
		18	MR-Egger	0.93 (0.57–1.53)	0.775	
		18	Weighted median	1.22 (0.93–1.61)	0.151	
		18	IVW	1.36 (1.13–1.64)	0.001	0.022
		18	Simple mode	2.02 (1.15–3.55)	0.025	
		18	Weighted mode	1.05 (0.68–1.64)	0.823	
Class *Lentisphaeria*	Sepsis					
		8	MR-Egger	0.79 (0.57–1.10)	0.211	
		8	Weighted median	0.85 (0.75–0.97)	0.016	
		8	IVW	0.86 (0.78–0.94)	0.002	0.026
		8	Simple mode	0.87 (0.71–1.07)	0.235	
		8	Weighted mode	0.89 (0.73–1.08)	0.273	
Order *Victivallales*	Sepsis					
		8	MR-Egger	0.79 (0.57–1.10)	0.211	
		8	Weighted median	0.85 (0.75–0.97)	0.015	
		8	IVW	0.86 (0.78–0.94)	0.002	0.033
		8	Simple mode	0.87 (0.71–1.08)	0.243	
		8	Weighted mode	0.89 (0.73–1.08)	0.266	

MR, Mendelian randomization; SNPs, number of single-nucleotide polymorphism. CI, confidence interval; OR, odds ratio; *p*_FDR_, *p*-value was calculated by the Benjamini–Hochberg method; LRTI, lower respiratory tract infection; IVW, inverse variance weighted.

### 3.2 Gut microbiota and pneumonia

Overall, nine gut microbiota were associated with the risk of respiratory infections in the primary MR analysis, suggesting that these gut microbiota may have an impact on the development of pneumonia. Among them, genus *Holdemanella* [OR:1.10, 95% confidence interval (CI): 1.03–1.19, *p* = 0.006] and genus *Oxalobacter* (OR: 1.09, 95% CI: 1.02–1.1.15, *p* = 0.005) were positively correlated with the risk of developing pneumonia. Class *Verrucomicrobiae* (OR: 0.88, 95% CI: 0.80–0.97, *p* = 0.009), order *Verrucomicrobiales* (OR: 0.88, 95% CI. 0.80–0.97, *p* = 0.009), family *Verrucomicrobiaceae* (OR: 0.88, 95% CI. 0.80–0.97, *p* = 0.009), genus *Akkermansi* (OR: 0.88, 95% CI: 0.80–0.97, *p* = 0.009), genus *ChristensenellaceaeR.7group* (OR: 0.83, 95% CI: 0.73–0.94, *p* = 0.005), genus *Coprococcus1* (OR: 0.89, 95% CI: 0.81–0.98, *p* = 0.020), and genus *RuminococcaceaeUCG002* (OR: 0.90, 95% CI: 0.83–0.98, *p* = 0.020) were negatively correlated with pneumonia (**Figure 2**). However, after BH correction, these genera were not associated with pneumonia.

### 3.3 Gut microbiota and URTI

In the primary MR analysis, seven gut microbiota were found to be associated with the risk of URTI. Among them, family *Defluviitaleaceae* (OR: 1.41, 95% CI:1.07–1.85, *p* = 0.014), genus *DefluviitaleaceaeUCG011* (OR: 1.44, 95% CI: 1.04–2.00, *p* = 0.027), genus *Erysipelatoclostridium* (OR: 1.28, 95% CI: 1.02–1.59, *p* = 0.030), and genus *Veillonella* (OR: 1.51, 95% CI: 1.03–2.23, *p* = 0.036) were positively associated with the risk of URTI, while class *Clostridia* (OR: 0.62, 95% CI: 0.44–0.86, *p* = 0.004), genus *Alistipes* (OR: 0.69, 95% CI: 0.51–0.93, *p* = 0.015), and genus *Streptococcus* (OR: 0.75, 95% CI: 0.57–0.98, *p* = 0.038) were negatively associated with the risk of URTI (**Figure 2**). None of these seven gut microbiota were associated with significance in URTI after BH correction.

### 3.4 Gut microbiota and LRTI

Nine gut microbiota were associated with the risk of LRTI (**Figure 2**). However, only three gut microbiota were associated with significance in LRTI after strict BH correction (**Table 1**). Specifically, we observed that the abundance of class *Coriobacteriia* (OR: 1.29, 95% CI: 1.12–1.48, *p*_FDR_ = 0.005), order *Coriobacteriales* (OR: 1.29, 95% CI: 1.12–1.48, *p*_FDR_ = 0.007), and family *Coriobacteriaceae* (OR: 1.29, 95% CI = 1.12–1.48, *p*_FDR_ = 0.011) were associated with a higher risk of LRTI.

**Figure 3:**
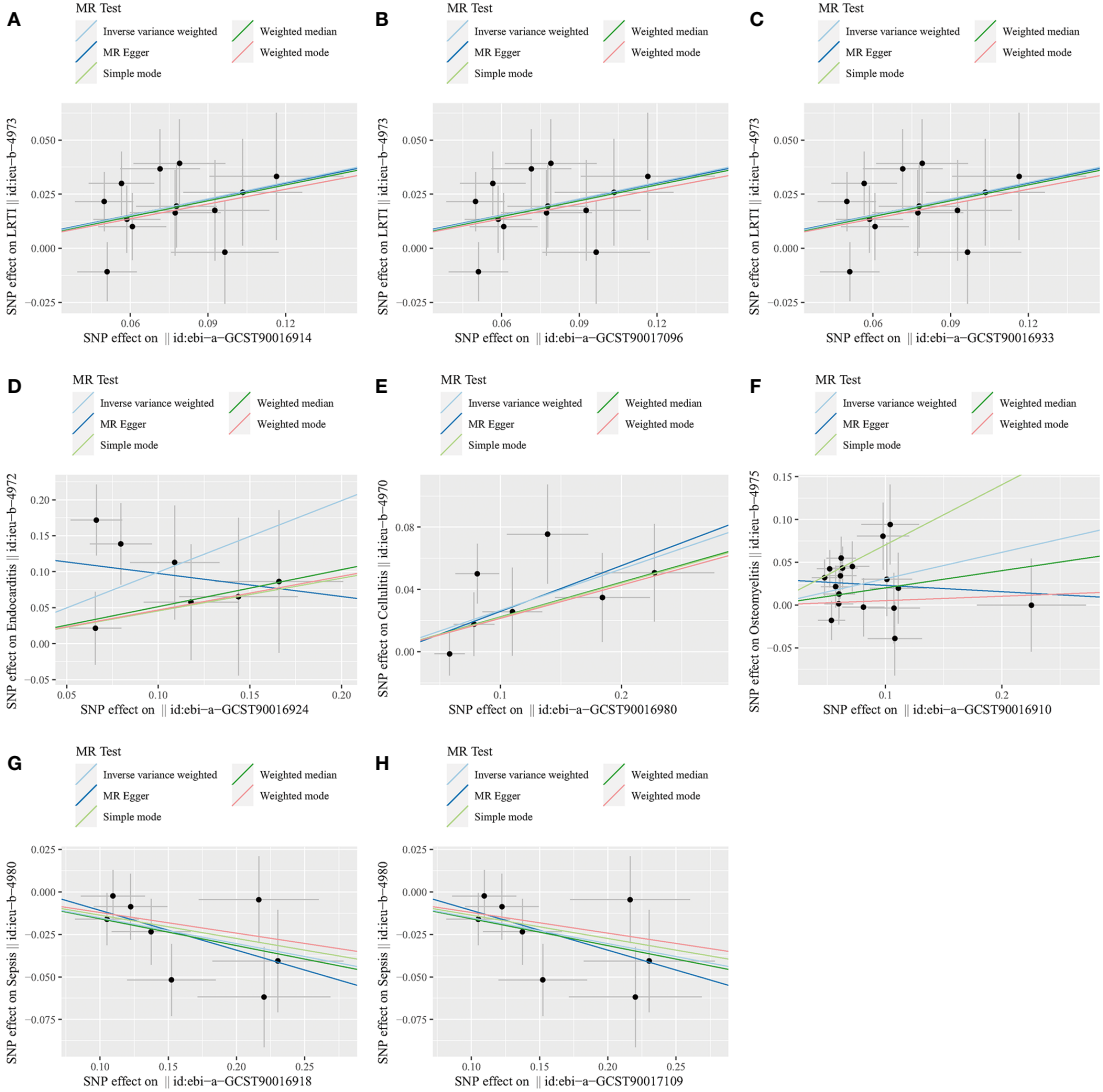
Scatter plots for the causal association between gut microbiota and infectious diseases. **(A)** Class *Coriobacteriia* and LRTI. **(B)** Order *Coriobacteriales* and LRTI. **(C)** Family *Coriobacteriaceae* and LRTI. **(D)** Family *Acidaminococcaceae* and endocarditis. **(E)** Genus *Clostridiumsensustricto1* and cellulitis. **(F)** Class *Bacilli* and osteomyelitis. **(G)** Class *Lentisphaeria* and sepsis. **(H)** Order *Victivallales* and sepsis. LRTI, lower respiratory tract infection.

In sensitivity analyses, the WME results were comparable to those of the IVW approach (OR: 1.28, 95% CI: 1.05–1.55, *p* = 0.012 for class *Coriobacteria*; OR: 1.28, 95% CI: 1.06–1.54, *p* = 0.010 for order *Coriobacteriales*; and OR: 1.28, 95% CI = 1.07–1.53, *p* = 0.007 for family *Coriobacteriaceae*), but with wider confidence intervals (**Figure 3**). Furthermore, the MR-Egger regression intercepts showed no evidence of pleiotropy of these gut microbiota with LRTI (intercept *p* = 0.977 for class *Coriobacteriia*; intercept *p* = 0.977 for order *Coriobacteriales*; and intercept *p* = 0.977 for family *Coriobacteriaceae*) (**Table 2** and **Supplementary Table S4**). No outliers were detected in the MRPRESSO regression. Heterogeneity analysis confirmed the accuracy of the results (**Table 2** and **Supplementary Table S5**). Data robustness was further validated by the leave-one-out results, showing a consistent positive association between gut flora and LRTI risk (**Supplementary Table S6**).

**Table 2:** Heterogeneity and sensitivity analysis between meaningful gut microbiota and infectious diseases.

Gut microbiota	Outcome	Methods	*Q*	*p*	Intercept	*p*	MR-PRESSO
Class *Coriobacteriia*	LRTI						
		IVW	7.998	0.785	0.001	0.977	0.927
		MR-Egger	7.997	0.714			
Order *Coriobacteriales*	LRTI						
		IVW	7.998	0.785	0.001	0.977	0.923
		MR-Egger	7.997	0.714			
Family *Coriobacteriaceae*	LRTI						
		IVW	7.998	0.785	0.001	0.977	0.929
		MR-Egger	7.997	0.714			
Family *Acidaminococcaceae*	Endocarditis						
		IVW	8.185	0.225	0.130	0.159	0.302
		MR-Egger	5.290	0.382			
Genus *Clostridium sensustricto1*	Cellulitis						
		IVW	5.574	0.473	-0.004	0.856	0.299
		MR-Egger	5.534	0.354			
Class *Bacilli*	Osteomyelitis						
		IVW	18.370	0.366	0.030	0.125	0.416
		MR-Egger	15.746	0.471			
Class *Lentisphaeria*	Sepsis						
		IVW	5.159	0.641	0.012	0.628	0.403
		MR-Egger	4.899	0.557			
Order *Victivallales*	Sepsis						
		IVW	5.159	0.641	0.012	0.628	0.394
		MR-Egger	4.899	0.557			

MR-PRESSO, Mendelian Randomization Pleiotropy RESidual Sum and Outlier; IVW, inverse variance weighted; LRTI, lower respiratory tract infection.

### 3.5 Gut microbiota and endocarditis

In the primary MR analysis, nine gut microbiota were associated with the risk of endocarditis (**Figure 2**). After BH correction, it was found that family *Acidaminococcaceae* abundance was positively associated with the risk of endocarditis (OR: 2.70, 95% CI: 1.47–4.97, *p*_FDR_ = 0.045) (**Table 1**).

In the sensitivity analysis, the WME method did not show statistical significance (OR: 1.67, 95% CI: 0.82–3.42, *p* = 0.159) (**Figure 3**). However, the MR-Egger regression intercept did not show evidence of multiplicity of family *Acidaminococcaceae* with endocarditis (Intercept *p* = 0.159) (**Table 2** and **Supplementary Table S4**). MRPRESSO regression did not detect outliers, too. The results of heterogeneity analysis confirmed the accuracy of the results (**Table 2** and **Supplementary Table S5**). The leave-one-out method further validated the data robustness (**Supplementary Table S6**).

### 3.6 Gut microbiota and UTI

Seven gut microbiota were confirmed to be associated with the risk of UTI after primary MR analysis. Among them, phylum *Euryarchaeota* (OR. 1.07, 95% CI: 1.02–1.13, *p* = 0.011), class *Bacteroidia* (OR: 1.11, 95% CI: 1.00–1.22, *p* = 0.044), order *Bacteroidales* (OR: 1.11, 95% CI: 1.00–1.22, *p* = 0.044), genus *Intestinibacter* (OR: 1.10, 95% CI: 1.00–1.20, *p* = 0.047), and genus *RuminococcaceaeUCG005* (OR: 1.12, 95% CI: 1.01–1.24, *p* = 0.025) were positively associated with the risk of UTI, while family *Defluviitaleaceae* (OR: 0.92, 95% CI: 0.84–1.00, *p* = 0.038) and genus *Defluviitaleaceae UCG011* (OR: 0.90, 95% CI: 0.82–0.99, *p* = 0.022) were negatively associated with the risk of UTI (**Figure 2**). No gut microbiota was causally associated with UTI after BH correction.

### 3.7 Gut microbiota and appendicitis

Primary MR analysis identified four gut microbiota associated with the risk of appendicitis. Among them, genus *LachnospiraceaeFCS020group* (OR: 1.32, 95% CI:1.09–1.61, *p* = 0.005) and genus *Turicibactera* (OR: 1.23, 95% CI: 1.01–1.50, *p* = 0.043) were positively associated with the risk of developing appendicitis, while family *Acidaminococcaceae* (OR: 0.73, 95% CI: 0.57–0.95, *p* = 0.017) and genus *Eisenbergiella* (OR: 0.86, 95% CI: 0.74–1.00, *p* = 0.045) were negatively associated with the risk of developing appendicitis (**Figure 2**). No gut microbiota was causally associated with appendicitis after BH correction.

### 3.8 Gut microbiota and cellulitis

Although 10 gut microbiota were associated with the risk of cellulitis (**Figure 2**), only genus *Clostridiumsensustricto1* was positively associated with cellulitis after BH correction (OR: 1.30, 95% CI: 1.13–1.55, *p*_FDR_ = 0.046) (**Table 1**).

In sensitivity analyses, the WME method showed similar results to IVW (OR: 1.25, 95% CI: 1.01–1.54, *p* = 0.036) (**Figure 3**). The MR-Egger regression intercept did not show evidence of multiplicity of genus *Clostridiumsensustricto1* with cellulitis (Intercept *p* = 0.856) (**Table 2** and **Supplementary Table S3**). MRPRESSO regression did not detect outliers. The results of heterogeneity analysis confirmed the accuracy of the results (**Table 2** and **Supplementary Table S5**). Meanwhile, leave-one-out results further validated the data robustness (**Supplementary Table S6**).

### 3.9 Gut microbiota and osteomyelitis

Seven gut microbiota were associated with the risk of osteomyelitis (**Figure 2**). However, only class *Bacilliidae* was positively causally associated with osteomyelitis after BH correction (OR: 1.36, 95% CI: 1.13–1.64, *p*_FDR_ = 0.022) (**Table 1**).

In sensitivity analyses, the WME method showed similar results to IVW (OR: 1.22, 95% CI: 0.93–1.61, *p* = 0.151) (**Figure 3**). The MR-Egger regression intercept did not show evidence of multiplicity of class *Bacilliidae* with cellulitis (Intercept *p* = 0.125) (**Table 2** and **Supplementary Table S3**). The MRPRESSO regression did not detect outliers. The results of heterogeneity analysis confirmed the accuracy of the results (**Table 2** and **Supplementary Table S5**). Meanwhile, leave-one-out results further validated the data robustness (**Supplementary Table S6**).

### 3.10 Gut microbiota and sepsis

We identified a total of 10 gut microbiota associated with sepsis (**Figure 2**); only 2 gut microbiota were associated with sepsis after BH correction (**Table 1**). Notably, class *Lentisphaeria* (OR: 0.86, 95% CI: 0.78–0.94, *p*_FDR_ = 0.026) and order *Victivallales* (OR: 0.86, 95% CI: 0.78–0.94, *p*_FDR_ = 0.033) abundance were negatively correlated with the risk of developing sepsis.

In the sensitivity analysis, the WME method showed similar results to IVW (OR: 0.85, 95% CI: 0.75–0.97, *p* = 0.016 for class *Lentisphaeria* and OR: 0.85, 95% CI: 0.75–0.97, *p* = 0.015 for order *Victivallales*) (**Figure 3**), and the MR-Egger regression intercept showed no evidence of pleiotropy (intercept *p* = 0.125 for class *Lentisphaeria* and intercept *p* = 0.944 for order *Victivallales*) (**Supplementary Table S3**). Heterogeneity analysis confirmed the accuracy of the results (**Table 2** and **Supplementary Table S5**). Leave-one-out results verified data robustness (**Supplementary Table S6**).

### 3.11 Inverse MR analysis

In the reverse MR, infectious disease was used as an exposure factor, and gut microbiota, which has been associated with infectious disease, was the outcome factor. The IVW results did not support a causal relationship between infectious disease and altered gut microbiota (**Supplementary Table 7**).

## 4 Discussion

In this study, TSMR was used to investigate the causal relationship between the relative abundance of gut microbiota and infectious diseases. It is currently believed that gut microbiota influences host metabolic health by producing a range of metabolites and molecules, including SCFA, bile acids, TMAO, and LPS. For instance, enterogenic SCFAs can affect the pulmonary immune environment in the respiratory system. Bacterial transmission, inflammation, and mortality increased when mice whose gut microbiota was disrupted by antibiotics developed pulmonary streptococcal infections. Furthermore, in mice with disrupted gut microbes, the alveolar macrophage metabolic pathway was upregulated, and the cellular response was altered, resulting in a reduced ability to phagocytize *S. pneumoniae*, causing a less pronounced immunomodulatory response (32). An imbalance of gut microbes can lead to damage to the intestinal wall, or “leaky gut.” A large number of toxins and bacteria enter the bloodstream through intestinal leakage to specific organs and tissues, thus triggering a series of inflammatory immune responses. Acute appendicitis is an intestinal infectious illness. Pathogenic bacteria multiply and secrete endotoxins and exotoxins, damaging the mucosal epithelium, forming ulcers, and allowing bacterial entry into the muscle layer of the appendix via the ulcerative surface. Increased interstitial pressure in the appendix wall affects arterial blood flow, resulting in appendicular ischemia and, in severe cases, infarction and gangrene (33). Infective endocarditis refers to the inflammation of the inner lining of the heart valve or ventricle caused by direct infection by bacteria, fungi, and other microorganisms. Studies have shown that intestinal flora destroys the intestinal mucosal barrier, and *Enterococcus faecalis* are released into the blood to attach to the normal valve and cause endocarditis (34). The main pathogen of cellulitis is hemolytic streptococcus, which is caused by external invasion of subcutaneous tissue or caused by lymphatic and hematologic infection (35). The interaction between intestinal flora and susceptibility to recurrent urinary tract infections (rUTI) may promote intestinal colonization of uropathogenic *Escherichia coli* (UPEC) through intestinal flora dysregulation and increase the risk of bladder infection. Furthermore, intestinal flora has been reported as an instigator, and its imbalance may cause systemic inflammation, further worsening the inflammation and symptoms after bladder infection (36). Gut microbiota can release pro-inflammatory or anti-inflammatory mediators and cytokines to regulate systemic bone metabolism through blood circulation. Studies have shown that gut microbiota disturbances that upregulate pro-IL1βlevels indirectly affect osteomyelitis (37).The occurrence and development of sepsis are closely related to the imbalance of gut microbiota. The disturbance of gut microbiota can induce sepsis through the destruction of intestinal mucosal barrier function, mucosal immune function, and bacterial translocation. At the same time, sepsis can also aggravate the imbalance of intestinal flora, resulting in multiple organ dysfunction (38).

Our study identifies a causal link between gut microbiota and infectious diseases, particularly that the abundance of class *Coriobacteriia*, order *Coriobacteriales*, and family *Coriobacteriaceae* are positively associated with the risk of LRTI. *Coriobacteriia* can be found in the mouth, respiratory tract, gastrointestinal tract, and reproductive tract. In the gut, class *Coriobacteriia* performs important functions such as the conversion of bile salts and steroids and the activation of dietary polyphenols. However, they can also be regarded as pathological diseases. According to previous research, the abundance of class *Coriobacteriia* can increase the incidence of diseases such as allergic rhinitis and endometriosis (39, 40). Family *Acidaminococcaceae*, genus *Clostridiumsensustricto1*, and class *Bacilli* were positively related to the risk of endocarditis, cellulitis, and osteomyelitis, respectively. Family *Acidaminococcaceae* belongs to strictly anaerobic Gram-negative coccus. Amino acids, especially glutamate, are a major source of energy (41). Genus *Clostridiumsensustricto1* belongs to Gram-positive bacterium fusobacterium; in the case of hypoxia, fusobacterium causes serious infections including tetanus and gas gangrene (42). Class *Bacilli* can bind lipopolysaccharide (LPS) and neutralize endotoxin. Therefore, the microecological preparation prepared by *Bacilli* has played an important role in the treatment of intestinal flora disorders and *Candida* infection (43). However, *Bacillus* cereus strains usually cause local wound and eye infection and systemic diseases (44). At the same time, the increased abundance of class *Lentisphaeria* and order *Victivallales* decreased the risk of sepsis. Surprisingly, *Lentisphaerae* has been reported to be more abundant in cases of inflammatory bowel disease (45) and less abundant in patients with sepsis, which is consistent with our conclusions (46). Order *Victivallales* has important effects on human infection and immune development. Specifically, it was found to be positively associated with clinical response to anti-programmed cell death protein-1 (PD-1) immunotherapy in patients with advanced cancer (47). In this regard, we believe that these gut microbiota may play a role in the occurrence and development of infectious diseases by regulating immunity. Interestingly, the findings of the reverse MR study do not support a causal relationship between infectious diseases and changes in gut microbiota.

One of the strengths of this study is that it established a causal relationship between alterations in gut microbiota and infectious diseases, offering candidate gut microbiota for subsequent functional studies. However, the study also has limitations. First, it only used European population GWAS data for TSMR analysis, and the abundance of gut microbiota included herein is limited, GWAS data of other gut microbiota need to be obtained in the future, to explore the causal relationship between gut microbiota and infectious diseases more comprehensively. Second, we did not further validate these results with public or our own datasets. Third, although TSMR is an efficient method of causality analysis, animal tests should be conducted in the future to further verify whether there is a potential causal relationship between gut microbiota and infectious diseases. Fourth, there are few studies on these gut flora that have causal relationship with infectious diseases, and more extensive studies are needed to support our conclusions in the future. Fifth, the causal relationship between gut microbiota and infectious diseases is multifaceted, necessitating the exploration of the etiology and pathogenesis of infectious diseases from multiple perspectives.

In conclusion, we used TSMR to explore the causal relationship between gut microbiota and infectious diseases. The results showed that the abundance of class *Coriobacteriia*, order *Coriobacteriales*, and family *Coriobacteriaceae* was associated with LRTI risk; family *Acidaminococcaceae*, genus *Clostridiumsensustricto1*, and class *Bacilli* were found to be positively related to the risk of endocarditis, cellulitis, and osteomyelitis, respectively. At the same time, the increased abundance of class *Lentisphaeria* and order *Victivallales* lowered the risk of sepsis. These findings elucidate the involvement of gut microbiota in the development of infectious diseases and offer a reference value for the treatment of infectious diseases.

## Data availability statement

The original contributions presented in the study are included in the article/**Supplementary Material**. Further inquiries can be directed to the corresponding authors.

## Ethics statement

Since the data used are publicly available in the database, no additional ethical approval was needed in this case.

## Author contributions

SW: Conceptualization, Data curation, Formal analysis, Visualization, Writing – original draft. FY: Investigation, Methodology, Resources, Writing – review & editing. WS: Software, Validation, Writing – original draft. RL: Methodology, Visualization, Writing – original draft. ZG: Formal Analysis, Writing – original draft. YW: Data curation, Writing – original draft. YZ: Conceptualization, Methodology, Writing – original draft. CS: Writing – original draft, Funding acquisition. DS: Conceptualization, Funding acquisition, Project administration, Writing – original draft.

## Supplementary material

Supplementary Table 1 Information on exposure and outcome factors.

Supplementary Table 2 Effect estimates of the associations between 196 bacterial traits and risk of infectious diseases in MR analyses.

Supplementary Table 3 The F number and *R* ^2^ detect the intensity of the IVs between 196 bacterial traits and risk of infectious diseases in MR analyses.

Supplementary Table 4 MR-Egger regression analysis between 196 bacterial traits and risk of infectious diseases in MR analyses.

Supplementary Table 5 Testing for heterogeneity between 196 bacterial traits and risk of infectious diseases in MR analyses.

Supplementary Table 6 Leave one out between 196 bacterial traits and risk of infectious diseases in MR analyses.

Supplementary Table 7 Effect estimates of the associations between infectious diseases and risk of nine infectious diseases in the reverse MR analyses.

Supplementary Table 8 Code required during data processing.
