# Supplementary material for: The causal relationship between gut microbiota and nine infectious diseases: a two-sample Mendelian randomization analysis
Source: Front Immunol. 2024 Jul 10;15:1304973. doi: 10.3389/fimmu.2024.1304973 (PMC11266007; doi:10.3389/fimmu.2024.1304973)
Supplement: Supplementary Table 1 — Information on exposure and outcome factors. [file Table_1.docx]

| **Type** | **Database source** | **Ancestry** | **Cases** | **Controls** | **Population** |
| --- | --- | --- | --- | --- | --- |
| Pneumonia | ieu-b-4976 | European | 22,567 | 463,917 | 486,484 |
| URTI | ieu-b-5063 | European | 2,795 | 483,689 | 486,484 |
| LRTI | ieu-b-4973 | European | 14,135 | 472,349 | 486,484 |
| Endocarditis | ieu-b-4972 | European | 1,080 | 485,404 | 486,484 |
| UTI | ieu-b-5065 | European | 21,958 | 464,256 | 486,214 |
| Appendicitis | ieu-b-4967 | European | 4604 | 481,880 | 486,484 |
| Cellulitis | ieu-b-4970 | European | 12,196 | 474288 | 486,484 |
| Osteomyelitis | ieu-b-4975 | European | 4,836 | 481,648 | 486,484 |
| Sepsis | ieu-b-4980 | European | 11,643 | 474,841 | 486,484 |
| Gut microbiota | FinnGen database | European |  |  | 1,531 |

**Table S1. Information on exposure and outcome factors.** **Abbreviations: URTI, Upper respiratory tract infection;LRTI, Lower respiratory tract infection;UTI, Urinary tract infection.**
