# Supplementary material for: The causal relationship between gut microbiota and nine infectious diseases: a two-sample Mendelian randomization analysis
Source: Front Immunol. 2024 Jul 10;15:1304973. doi: 10.3389/fimmu.2024.1304973 (PMC11266007; doi:10.3389/fimmu.2024.1304973)
Supplement: Supplementary Table 8 — Code required during data processing. [file DataSheet_1.docx]

library(TwoSampleMR)

library(data.table)

library(tidyverse)

library(readxl)

library(writexl)

name <- read.table("1.txt")

exp_dat_ids <- name[,1]

exps <- name[,1]

outcomeid <- c("ID")

#

dir.create(path = "mendelian")

for (qaq in 1:length(exp_dat_ids)) {{ #

exp_dat_id <- exp_dat_ids[qaq]

exp <- exps[qaq]

exp_data <- extract_instruments(

outcomes = exp_dat_id,p1=1e-05,clump =TRUE )

if(length(exp_data[,1])>2){

out_data <- extract_outcome_data(

snps = exp_data$SNP,

outcomes = outcomeid)

dat <- TwoSampleMR::harmonise_data(

exposure_dat = exp_data,

outcome_dat = out_data)

dat <-subset(dat,mr_keep==TRUE)

snp_add_eaf <- function(dat, build = "37", pop = "EUR")

{

stopifnot(build %in% c("37","38"))

stopifnot("SNP" %in% names(dat))

# Create and get a url

server <- ifelse(build == "37","http://grch37.rest.ensembl.org","http://rest.ensembl.org")

pop <- paste0("1000GENOMES:phase_3:",pop)

snp_reverse_base <- function(x)

{

x <- str_to_upper(x)

stopifnot(x %in% c("A","T","C","G"))

switch(x,"A"="T","T"="A","C"="G","G"="C")

}

res_tab <- lapply(1:nrow(dat), function(i)

{

print(paste0("seaching for No.", i, " SNP"))

dat_i <- dat[i,]

ext <- paste0("/variation/Homo_sapiens/",dat_i$SNP, "?content-type=application/json;pops=1")

url <- paste(server, ext, sep = "")

res <- httr::GET(url)

# Converts http errors to R errors or warnings

httr::stop_for_status(res)

# Convert R objects from JSON

res <- httr::content(res)

res_pop <- jsonlite::fromJSON(jsonlite::toJSON(res))$populations

# Filter query results based on population set

res_pop <- try(res_pop[res_pop$population == pop,])

if("try-error" %in% class(res_pop))

{

print(paste0("There is not information for population ",pop))

queried_effect_allele <- "NR"

queried_other_allele <- "NR"

queried_eaf <- -1

}

else

{

if(nrow(res_pop)==0)

{

print(paste0("There is not information for population ",pop))

queried_effect_allele <- "NR"

queried_other_allele <- "NR"

queried_eaf <- -1

}

else

{

queried_effect_allele <- res_pop[1,"allele"][[1]]

queried_other_allele <- res_pop[2,"allele"][[1]]

queried_eaf <- res_pop[1,"frequency"][[1]]

}

}

effect_allele <- ifelse("effect_allele.exposure" %in% names(dat),

dat_i$effect_allele.exposure,

dat_i$effect_allele)

other_allele <- ifelse("effect_allele.exposure" %in% names(dat),

dat_i$other_allele.exposure,

dat_i$other_allele)

if("effect_allele.exposure" %in% names(dat))

{

name_output <- unique(c(names(dat), "eaf.exposure","reliability.exposure"))

}

else

{

name_output <- unique(c(names(dat), "eaf","reliability.exposure"))

}

len_effect_allele <- nchar(effect_allele)

len_other_allele <- nchar(other_allele)

if(len_effect_allele==1&len_other_allele==1)

{

if((queried_effect_allele==effect_allele & queried_other_allele==other_allele)|

(queried_effect_allele==other_allele & queried_other_allele==effect_allele))

{

dat_i$eaf.exposure <- ifelse(effect_allele == queried_effect_allele,

queried_eaf,

1-queried_eaf)

dat_i$eaf <- dat_i$eaf.exposure

dat_i$reliability.exposure <- "high"

}

else

{

r_queried_effect_allele <- snp_reverse_base(queried_effect_allele)

r_queried_other_allele <- snp_reverse_base(queried_other_allele)

if((r_queried_effect_allele==effect_allele & r_queried_other_allele==other_allele)|

(r_queried_effect_allele==other_allele & r_queried_other_allele==effect_allele))

{

dat_i$eaf.exposure <- ifelse(effect_allele == r_queried_effect_allele,

queried_eaf,

1-queried_eaf)

dat_i$eaf <- dat_i$eaf.exposure

dat_i$reliability.exposure <- "high"

}

else

{

dat_i$eaf.exposure <- ifelse(effect_allele == queried_effect_allele,

queried_eaf,

1-queried_eaf)

dat_i$eaf <- dat_i$eaf.exposure

dat_i$reliability.exposure <- "low"

}

}

}

else

{

# To identify the potential DEL/ INS

short_allele <- ifelse(len_effect_allele==1,

effect_allele,

other_allele)

short_allele_eaf <- ifelse(short_allele == queried_effect_allele,

queried_eaf,

1-queried_eaf)

dat_i$eaf.exposure <- ifelse(effect_allele == short_allele,

short_allele_eaf,

1-short_allele_eaf)

dat_i$eaf <- dat_i$eaf.exposure

dat_i$reliability.exposure <- "low"

}

dat_i[name_output]

})

return(do.call(rbind, res_tab))

}

dat <- snp_add_eaf(dat)

get_f<-function(dat,F_value=10){

log<-is.na(dat$eaf.exposure)

log<-unique(log)

if(length(log)==1)

{if(log==TRUE){

Print

return(dat)}

}

if(is.null(dat$beta.exposure[1])==T || is.na(dat$beta.exposure[1])==T){print

return(dat)}

if(is.null(dat$se.exposure[1])==T || is.na(dat$se.exposure[1])==T){print

return(dat)}

if(is.null(dat$samplesize.exposure[1])==T || is.na(dat$samplesize.exposure[1])==T){print

return(dat)}

if("FALSE"%in%log && is.null(dat$beta.exposure[1])==F && is.na(dat$beta.exposure[1])==F && is.null(dat$se.exposure[1])==F && is.na(dat$se.exposure[1])==F && is.null(dat$samplesize.exposure[1])==F && is.na(dat$samplesize.exposure[1])==F){

R2<-(2*(1-dat$eaf.exposure)*dat$eaf.exposure*(dat$beta.exposure^2))/((2*(1-dat$eaf.exposure)*dat$eaf.exposure*(dat$beta.exposure^2))+(2*(1-dat$eaf.exposure)*dat$eaf.exposure*(dat$se.exposure^2)*dat$samplesize.exposure))

F<- (dat$samplesize.exposure-2)*R2/(1-R2)

dat$R2<-R2

dat$F<-F

dat<-subset(dat,F>F_value)

return(dat)

}

}

dat <- get_f(dat, F_value = 10)

res=TwoSampleMR::mr(dat)

print(paste0(exp,"_SNP_",res$nsnp[1]))

results <- TwoSampleMR::generate_odds_ratios(res)

results$estimate <- paste0(

format(round(results$or, 2), nsmall = 2), " (",

format(round(results$or_lci95, 2), nsmall = 2), "-",

format(round(results$or_uci95, 2), nsmall = 2), ")")

resdata <- dat

openxlsx::write.xlsx(dat,file = paste0("mendelian/",exp,"-dat.xlsx"), row.names = FALSE)

names(resdata)

Assumption13 <- subset(resdata,mr_keep==TRUE,

select = c("SNP","pval.exposure",

"pval.outcome", # "F_statistic",

"mr_keep"))

res_hete <- TwoSampleMR::mr_heterogeneity(dat)

res_plei <- TwoSampleMR::mr_pleiotropy_test(dat)

res_leaveone <- mr_leaveoneout(dat) #

res_presso <- TwoSampleMR::run_mr_presso(dat,NbDistribution = 100)

# [["MR-PRESSO results"]][["Global Test"]][["Pvalue"]]

sink(paste0("mendelian/",exp,"_PRESSO.txt"),

append=FALSE,split = FALSE)

print(res_presso)

sink()

print(res_presso)

openxlsx::write.xlsx(x = list(

"main"=results,

"Assumption13"=Assumption13,

"pleiotropy"=res_plei,

"heterogeneity"=res_hete,

"leaveone"=res_leaveone),

overwrite = TRUE,

paste0("mendelian/",exp,"-res.xlsx"))

p1 <- mr_scatter_plot(res, dat)

p1[[1]]

pdf(paste0("mendelian/",exp,"_scatter.pdf"))

print(p1[[1]])

dev.off()

res_single <- mr_singlesnp(dat)

p2 <- mr_forest_plot(res_single)

pdf(paste0("mendelian/",exp,"_forest.pdf"))

print(p2[[1]])

dev.off()

p3 <- mr_funnel_plot(res_single)

pdf(paste0("mendelian/",exp,"_funnel.pdf"))

print(p3[[1]])

dev.off()

res_loo <- mr_leaveoneout(dat)

pdf(paste0("mendelian/",exp,"_leave_one_out.pdf"))

print(mr_leaveoneout_plot(res_loo))

dev.off()

}

library(magrittr)

res2 <- res[1:3,]

judge_1 <- function(mr_res=res2) {

mr_res$b_direction <- as.numeric(sign(mr_res$b))

mr_res$b_direction=ifelse(abs(sum(mr_res$b_direction))==3 ,

NA,"Inconsistent direction")

mr_res$p_no <- NA

mr_res[mr_res$method=="MR Egger","p_no"] <- ifelse(

mr_res[mr_res$method=="MR Egger","pval"]<0.05," ",

"MR Egger")

mr_res[mr_res$method=="Weighted median","p_no"] <- ifelse(

mr_res[mr_res$method=="Weighted median","pval"]<0.05," ",

"Weighted median")

mr_res[mr_res$method=="Inverse variance weighted","p_no"] <- ifelse(

mr_res[mr_res$method=="Inverse variance weighted","pval"]<0.05,

" ","Inverse variance weighted")

mr_res$p_no <- paste(mr_res$p_no,collapse = " ")

mr_res$p_no=trimws(mr_res$p_no,which = c("both"))

return(mr_res)

}

res3 <- judge_1(mr_res = res2)

library(magrittr)

# Main result

res4 <- tidyr::pivot_wider(

res3,names_from ="method",names_vary = "slowest",

values_from = c("b","se","pval") )

# Heterogeneity statistics

res_hete2 <- tidyr::pivot_wider(

res_hete,names_from ="method",names_vary = "slowest",

values_from = c("Q","Q_df","Q_pval") ) %>%

dplyr::select( -id.exposure,-id.outcome,-outcome,-exposure)

# Horizontal pleiotropy

res_plei2 <- dplyr::select(res_plei,

egger_intercept,se,pval)

# Merge

res_ALL <- cbind(res4, res_hete2, res_plei2)

write.csv(res_ALL,file = paste0("mendelian/",exp,".csv"), row.names = FALSE)

}}

fs=list.files("D:/mendelian", pattern = "csv",full.names = TRUE) #

df = map_dfr(fs, read.csv)

write.csv(df,"res.csv")
